# War and education: the attacks on medical schools amidst ongoing armed conflict, Sudan 2023

**DOI:** 10.1186/s13031-024-00584-7

**Published:** 2024-03-29

**Authors:** Esra Abdallah Abdalwahed Mahgoub, Amna Khairy, Samar Osman, Musab Babiker Haga, Sarah Hashim Mohammed Osman, Abubker Mohammed Abbu Hassan, Hala Kamal, Ayia Babiker

**Affiliations:** 1 Faculty of Medicine, International University of Africa, Khartoum, Sudan; 2 Eastern Mediterranean Region network for public health, Khartoum, Sudan; 3 Faculty of Dentistry, National University, Khartoum, Sudan; 4 Faculty of Dentistry, National Ribat University, Khartoum, Sudan; 5 Faculty of Medicine, University of Khartoum, Khartoum, Sudan; 6 Imperial College London, Royal Hospital for Children and Young People, Edinburgh, Scotland

**Keywords:** Sudan, War, Attacks, Medical Education, Medical School, Distance Education

## Abstract

**Background:**

War results in widespread destruction of a country’s infrastructure, healthcare facilities, and educational institutions. This study aims to assess the attacks on medical schools amidst the ongoing conflict in Sudan.

**Methods:**

A descriptive cross-sectional study was conducted across 58 medical schools located in the states of Khartoum, Darfur, and Kordofan. Data on attacks between April 15, 2023, and July 15th 2023, were collected using online data collection form.

**Results:**

All medical schools in conflict areas were included in the study. More than half (58.6%) of these medical schools were attacked. Private schools, constituting the majority of the study sample, were the most frequently attacked (70.6%). Of these, 52.9% were located in Khartoum city. More than one form of attack was reported in 64.7% of the affected schools. Looting occurred in 73.5% of the attacked faculties, while 67.6% of them were converted into military bases. Despite these challenges, 60.3% of the schools in the conflict zone managed to restore the educational process through online learning and collaboration with other institutions.

**Conclusion:**

During a three-month period of warfare, most medical schools within conflict zones were attacked. This emphasizes the vulnerability of medical education institutions during war and highlights the urgent need of the Ministry of Higher Education interventions to provide leadership, support, and oversight for the educational process in medical schools across the country.

**Supplementary Information:**

The online version contains supplementary material available at 10.1186/s13031-024-00584-7.

## Introduction

Formal medical education in Sudan began with the establishment of the Gordon School of Medicine in 1924 [1]. Investment in higher education resulted in the establishment of numerous public and private medical schools. Sudan now homes more than 70 medical schools and has an annual uptake exceeding 5,000 students [1], representing 23% of medical schools in Sub-Saharan Africa. In 2018, the Sudan Medical Council (SMC) received recognition from the World Federation of Medical Education, making it the 10th accrediting agency to receive this validation, a step toward continuing to enhance the country’s medical education quality [2]. Although the medical education system in Sudan was on an upward trajectory, the healthcare system remained fragile. Achieving equitable coverage of Human Resources for Health (HRH) has been a long-standing challenge in Sudan. In 2021, the Sudanese physician-to-patient ratio was limited to 2.1 physicians per 10,000 population, significantly lower than the recommended health worker density of 134 per 10,000 population needed to achieve 70% or more universal health coverage in Africa [3, 4]. Sudan has suffered a crippling shortage of doctors following decades of healthcare worker migration, documented since the 1960s and continuing today [5].

The recent severe instability in Sudan has led to inadequate resource allocation in the country’s medical education and healthcare [6]. Following the outbreak of war on the 15th of April 2023 between Sudanese military and paramilitary groups, the country’s infrastructure has been significantly damaged, including hospitals, public buildings, and humanitarian offices/assets, leaving a trail of destruction. The resultant deterioration of the healthcare landscape has brought with it a catastrophic humanitarian and economic crisis [7]. During this period of conflict, the Sudanese people have experienced restrictions in movement alongside inaccessibility to education, human rights that are essential for the stability and development of any country.

Given the difficulties associated with conducting on-site assessments regarding the status of medical schools within conflict zones, there remains limited understanding of the immediate impact and damage of war on these institutions. Thus, this study used an online survey of medical schools in conflict-affected regions of Sudan to assess the occurrence and impact of attacks on these educational establishments to bridge this knowledge gap and provide relevant authorities with information to facilitate informed decision-making regarding the continuity of the education process.

## Materials and methods

### Study design, population, and area

**Fig. 1:**
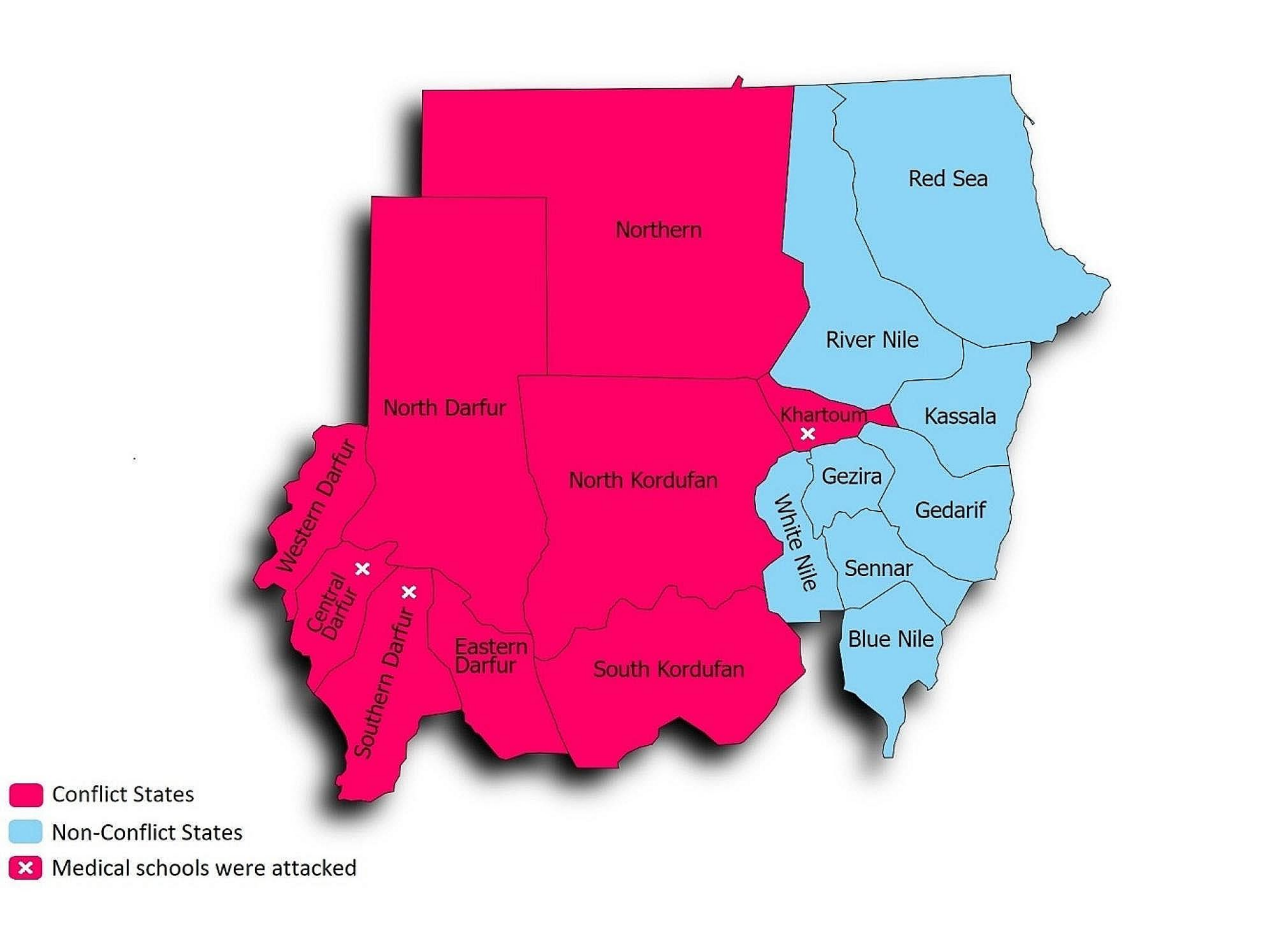
The conflict states included in the study

A descriptive cross-sectional study was conducted across medical schools located in the areas affected by the current war in Sudan, which started on April 15th 2023. The study included all medical faculties located in Meroe City and the states of Khartoum, Darfur (Central, East, North, South and West Darfur), and Kordofan (North and South Kordofan). Figure 1 illustrates the conflict states included in the study.

Total coverage was employed to include all medical schools in the targeted states. This amounted to a total of 58 medical faculties. The schools were identified using the Admission guide for Sudanese universities 2023, published by the country’s Ministry of Higher Education and Scientific Research.

### Data collection

Quantitative data were collected via author-designed, structured data collection form (Google form) that gathered information about the university type (i.e., public, private or military affiliated), its location, whether it was subjected to any kind of violations, the type of attacks (destruction of the building, burning or setting fire, looting or stealing, conversion into a military base, or detention) and size of the damage sustained, and whether the faculty had implemented measures to resume the study during this period.

The forms were filled out by six data collectors using the following resources:

1. Extensive web search involving the official institutions’ websites, the official institution’s Facebook and X (Twitter) pages, Students’ association Facebook and X pages, news pages, and posts on Facebook and X (Twitter) websites. This was done using a predefined bilingual (Arabic and English) search strategy including the following terms: Names of the universities, Medical Universities, Medical Schools, Medical Education, Attacks; War; Conflict; Armed Conflict, Military Conflicts, Looting; Destruction, Damage; Sudanese Armed Forces; Rapid Support Forces, SAF, RSF, Sudan.
2. Personal communication with administrative university staff, academic staff, students, and eyewitnesses. They were contacted through phone calls or social media applications.

To gather comprehensive data and ensure its accuracy, the data collectors were required to initially search all available online sources. If the data was not found in these sources or was available in news pages or social media posts only, they then had to validate the data through direct communication with the faculties administrative and/or academic staff. If further information was needed or if contact with staff was unsuccessful, they would then seek input from students or eyewitnesses.

The data was deemed highly valid if it originated from multiple sources or from any of the following sources: the University Official website, University Facebook/Twitter pages, university administration staff, or Student Association Facebook/Twitter pages. Conversely, data from a single source, such as social media posts/comments, academic staff, students or eyewitnesses, or news pages, was considered to have low validity.

### Statistical analysis

Quantitative data from the data collection form were extracted into an Excel spreadsheet. Analysis was performed using a Statistical Package for the Social Sciences version 26. Data were instead presented in a descriptive manner. Frequencies and percentages about the faculties’ characteristics (Type, and geographical location), characteristics of attacks on medical faculties, and methods of continuance of education in faculties are presented using tables and bar charts. No inferential statistical analysis was conducted.

## Results

### Type and location of medical faculties

A total of 58 medical faculties were included in our study. Of these, 70.7% (41/58) were private, and the majority (75.9%, 44/58) were in Khartoum state (see Table 1 for further details on the type and location of the faculties included.)

**Table 1:** The faculties’ characteristics (*n* = 58)

		n	%
Type of the school	Private	41	70.7
	Public	15	25.9
	Related to military forces	2	3.4
Geographical distribution of the faculties	Khartoum State	44	75.9
	Kordofan States	5	8.6
	Darfur States	8	13.8
	Northern State (Marawi)	1	1.7

### Characteristics of attacks on medical faculties

**Fig. 2:**
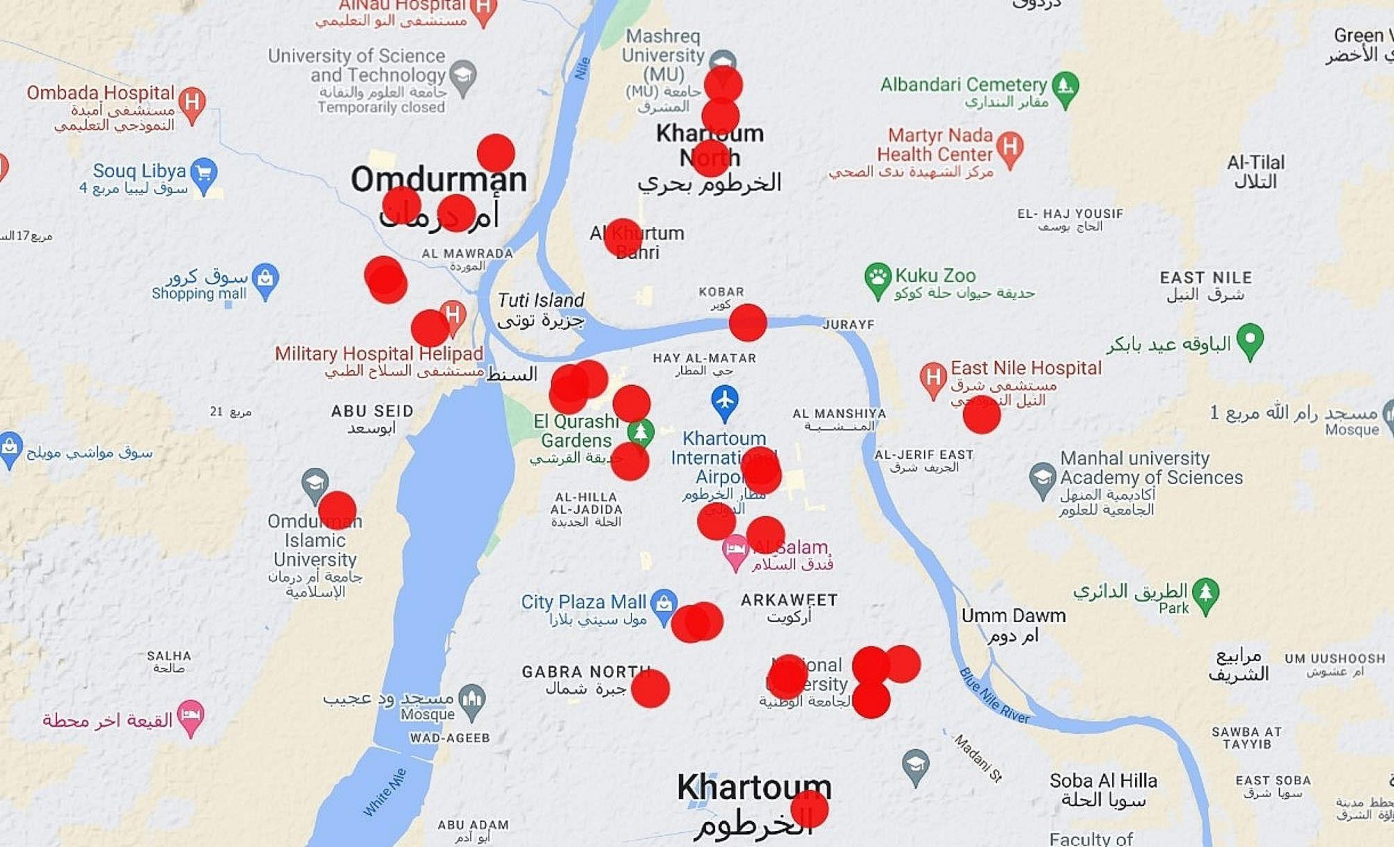
The location of the attacked medical schools in Khartoum State

More than half of the medical faculties within the conflict areas were attacked (58.6%, 34/58). We could not obtain information from five faculties due to poor network connections in their area or an absence of information about the university being on an active battlefield. These five faculties were, however, included in the analysis as part of the denominator. Of the attacked faculties, three-quarters were private (70.6%, 24/34), with 52.9% (18/34) in Khartoum city and 20.6% (7/34) in Omdurman city (Fig. 2).

**Fig. 3:**
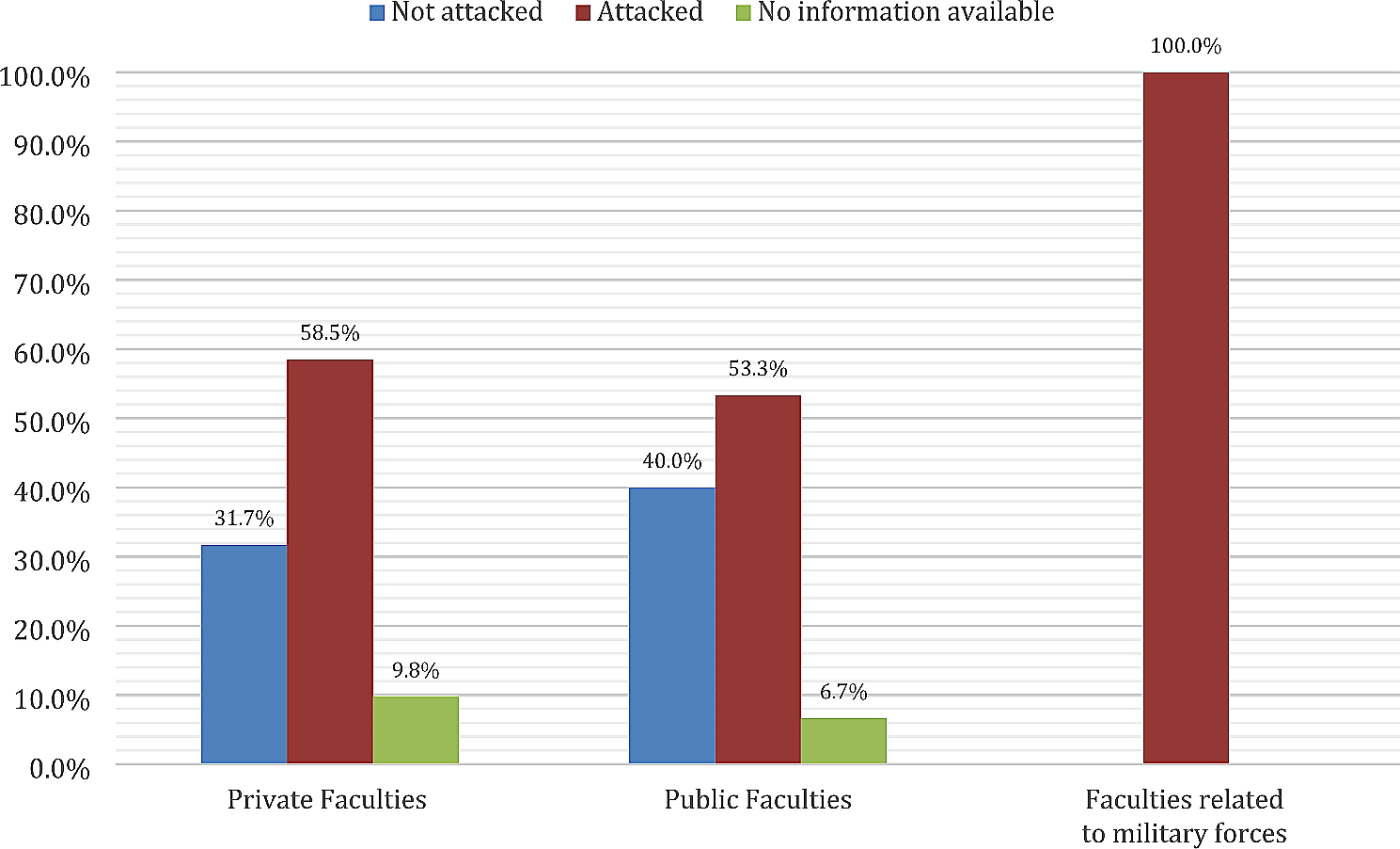
Frequency of attacks in different types of faculties (*n* = 58)

Furthermore, 64.7% (22/34) of schools suffered more than one form of attack compared to 35.3% (12/34) suffering a single. The majority of faculties (73.5%, 25/34) were looted, and 67.6% (23/34) were converted into military bases. In one university, the students were detained in the university dorm for more than one week before diplomatic efforts released them. The two faculties with military affiliations (one faculty established by and affiliated with the Sudanese Armed Forces and the other with the Sudan Police Force) were converted into military bases, and 58.5% (24/41) of the private faculties were attacked, compared to 53.3% (8/15) of the public faculties (Fig. 3).

In 41.2% (14/34) of the faculties, the administrative buildings were attacked, whereas laboratories and lecture halls were attacked in 20.6% (7/34) and 14.7% (5/34), respectively.

Information regarding attacks was obtained from multiple sources in 41.2% (14/34) of the cases and from a single source in 58.8% (20/34). Social media posts/comments, personal communication (with students, academic staff, or eyewitnesses), and administration staff of the faculties were the three top sources of information about the attacks. Furthermore, 70.6% [24] of the data was considered to have high validity. More details about the attacks are provided in Table 2.

**Table 2:** Characteristics of attacks on medical faculties

Variable	Items	n	%
Was the university attacked during the ongoing war? (*n* = 58)	Yes	34	58.6
	No	19	32.8
	No information available	5	8.6
Location of attacked universities by city (*n* = 34)	Khartoum	18	52.9
	Bahri	6	17.6
	Omdurman	7	20.6
	Nayala	2	5.9
	Zalingei	1	2.9
Type of the school (*n* = 34)	Private	24	70.6
	Public	8	23.5
	Related to military forces	2	5.9
Type of attack	Destruction of the building	16	47.1
	Burning or setting fire	4	11.8
	Looting or stealing	25	73.5
	Conversion into a military base	23	67.6
	Detention	1	2.9
Extent of damage	Laboratories	7	20.6
	Lecture halls	5	14.7
	Administrative buildings	14	41.2
	Student’s support services buildings	7	20.6
	University walls and squares	3	8.8
	No information about the extent of the damage	13	38.2
Source of information	Social media posts/comments	15	44.1
	Academic Staff/students/eyewitnesses	13	38.2
	Administration staff of the university	9	26.5
	Student Association Facebook/Twitter pages	6	17.6
	University Facebook/Twitter pages	5	14.7
	News-page	4	11.8
	University Official website	1	2.9
Validity of the data according to the source	High	24	70.6
	Low	10	29.4

### Continuation of the educational process

Attempts at restoring educational processes were demonstrated by 60.3% (35/58) of the included faculties (21 attacked and 14 not attacked faculties). This was through online education in 48.6% (17/35), collaboration with other universities both outside and inside Sudan in 8.6% (3/35), or both strategies (e-learning and collaboration) in 40% (14/35) of schools. Only one not attacked university was able to continue teaching on campus (Supplementary Table 1, Additional File 1).

**Fig. 4:**
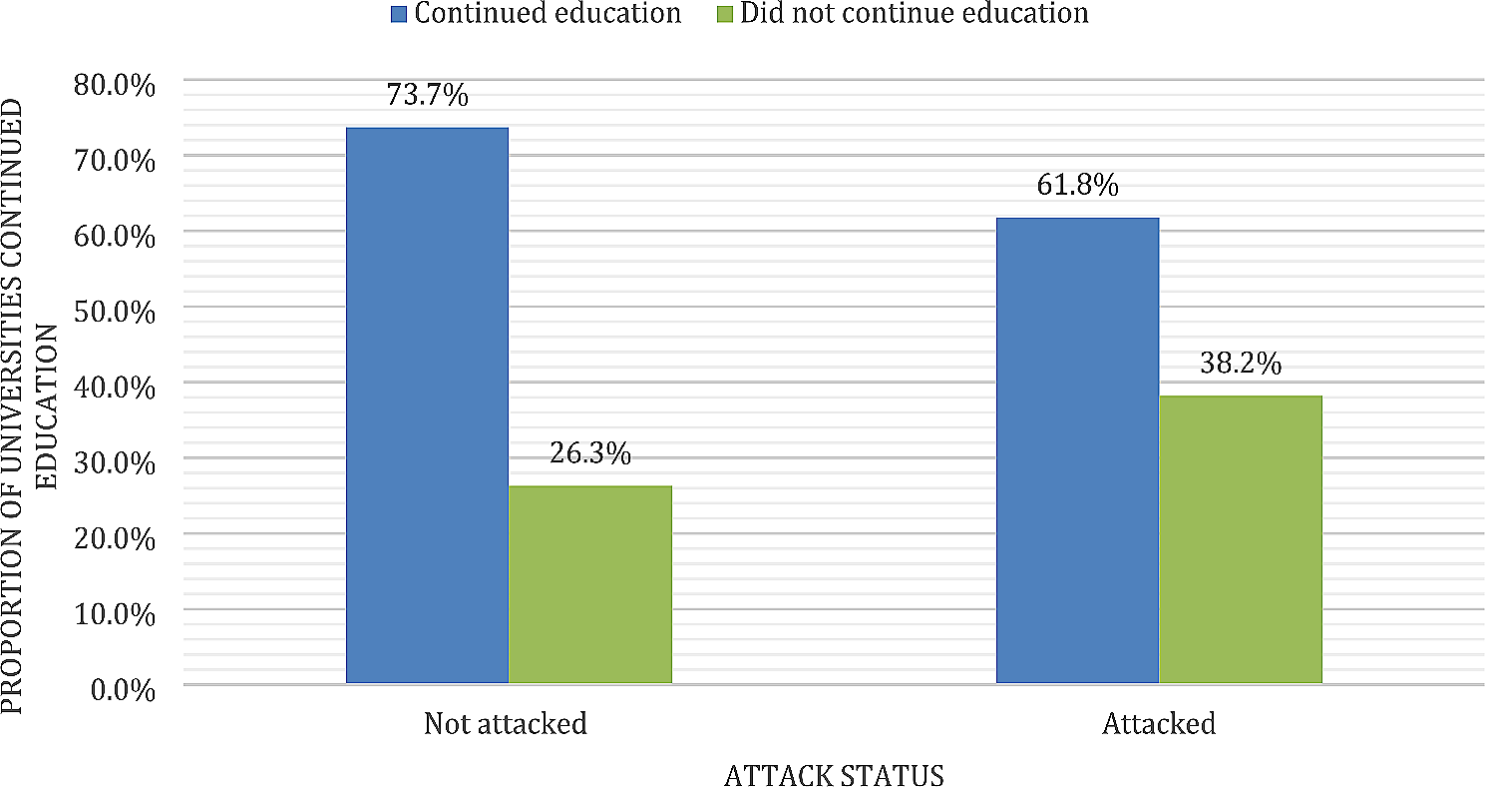
Frequency of educational process continuation according to the attack status (*n* = 58)

**Fig. 5:**
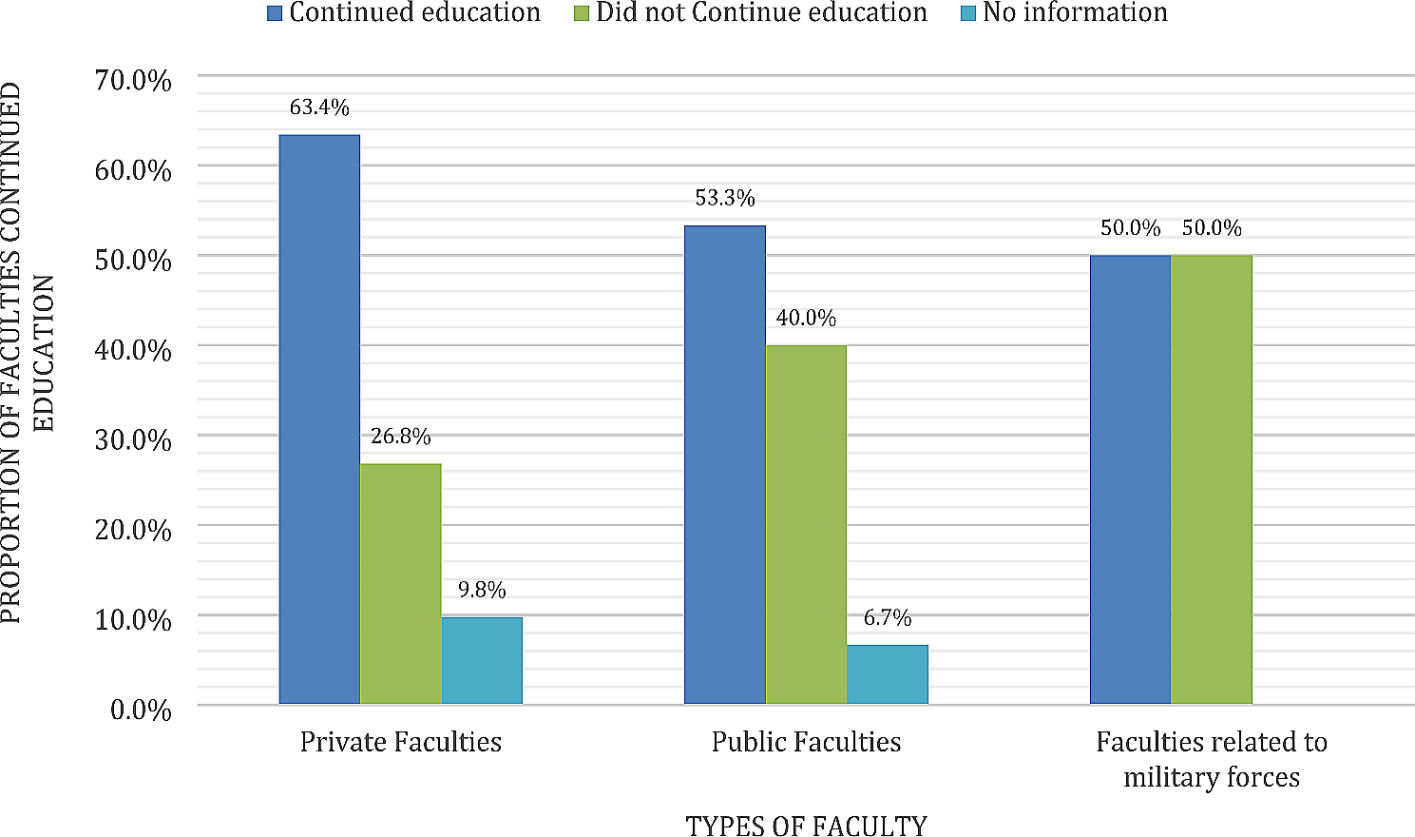
Frequency of continuance of education in different types of faculties (*n* = 58)

Interestingly, 61.8% of the attacked universities were able to restore educational processes, compared to 73.7% of the universities that were not attacked (Fig. 4). Looking at this based on type of institution, education was restored in 63.4% (26/41) of the private faculties, 53.3% (8/15) of public faculties, and 50% (1/2) of faculties related to the military (Fig. 5).

## Discussion

This cross-sectional study sheds light on the alarming attacks faced by medical schools amidst the ongoing conflict in Sudan. The findings indicate that more than half of the medical faculties located in conflict zones have been subjected to attacks. Despite challenges, approximately 60.3% of the schools were able to restore some educational processes through measures such as online education and collaboration with other universities both within and outside the country.

Attacks on medical educational institutions during times of war have been observed in various countries, including Liberia, Iraq, Croatia, and others [8–10]. These assaults breach international humanitarian law, particularly the Fourth Geneva Convention and the Hague Convention for the Protection of Cultural Property in the Event of Armed Conflict [11, 12]. These conventions forbid the targeting and utilization of civilian objects for military ends, which can be applied to educational institutions [11, 12]. During the first three months of the April 15 war in Sudan, more than half of the medical faculties within the conflict zone were attacked. The administrative buildings were affected in 41.2% of cases, risking loss of vital documentation systems, student records, and college servers. University data were particularly precarious if not stored on an online storage system or cloud. The lack of security at faculties resulted in their looting, a finding similar to reports from the Afghanistan war [13]. As witnessed in Liberia and Syria, this warfare destruction will have long-term consequences, disrupting the educational journeys of hundreds of students, denying their right to education, and inevitably delaying their medical careers [8, 14].

Interestingly, out of the 58 schools affected by the war, 35 managed to resume their educational activities. Amidst the Ukraine war, universities shifted to online learning, resulting in the suspension of clinical training in most cases [15]. Comparatively, in Sudan, 17 universities were able to continue providing education via collaborations with other universities. This involved relocating students to safer regions for clinical training or partnering with foreign universities and transferring students’ entire educational process. However, restoring clinical training in Sudan through student relocation is a costly process. Given the recent spike in the cost of living in Sudan, specifically housing prices in the safe regions in the country, the financial burden relocation brings with it to both the institution and its students renders this option unfeasible to many. In addition, the destruction of buildings and hospitals results in inadequate training infrastructures, and the significant impact of healthcare worker brain drain on medical training poses significant hurdles to overcome [16–18].

The utilization of online education to reinstate educational processes has been employed by 31 faculties. E-learning was first trialled in Sudan during the COVID-19 pandemic. This initial roll out highlighted various obstacles to its implementation in the country. Most notably, inadequate network infrastructure, limited bandwidth in rural areas, power outages, and the absence of electricity in rural regions presented significant barriers [19, 20]. Electricity availability and network quality deteriorated further during the war. Recent reports indicate frequent power outages and network disruptions due to unreliable power supplies and an inability to fuel generators by service providers [21, 22]. This presents a major obstacle in the adoption of online education, affecting its accessibility and acceptability among both students and staff, as well as its inclusion of all students in the learning process. Therefore, further investigation is required to determine if online learning can be a successful educational approach in this complex context.

The ongoing conflict in Sudan has had a detrimental impact on the country’s already fragile economy, making it challenging to rebuild the damaged infrastructure. This is compounded by the fact that Sudan allocates a low percentage of its public expenditure (2.1%) to education [23]. Overcoming these financial challenges would involve key stakeholders of the higher education sector reallocating and mobilizing resources toward continuing education through the prioritization of system rebuilding and preventing resource diversion toward military purposes [10]. The majority of the targeted faculties were private institutions, increasing pressure on investors to find innovative solutions and ensure that students receive a quality education that equips them with the necessary skills to become competent doctors. During similar conflicts, a study conducted in Iraq highlighted a decline in the quality of education [24]. This underscores the crucial role that the Ministry Higher Education can exert in overseeing the educational process during these challenging times to maintain educational standards.

Medical school attacks present an unforeseen threat to HRH availability, a long-term consequence of the conflict. Despite the measures taken by medical universities toward training continuation, these fail to adequately respond to local health needs over the short and long term. However, collaboration with other universities in states not affected by the conflict could positively impact the accessibility, quality, and equity of HRH coverage at subnational levels.

This study also highlights a gap in coordinated HRH planning, education, and leadership at the national level. All efforts toward mitigating the impact of conflicts identified through this study were solely led by the universities. We were unable to identify any evidence of coordination on a national scale from either the ministries of higher education or health. This was reflected in the lack of reports on university attacks from both formal governing bodies. A coordinated approach is critical to ensure that HRH is responsive to current and emerging local health needs [25]. To ensure effective and efficient distribution of resources, an approach to link both the public and private sectors for health and education should be implemented.

### Limitations

Considering the limited accuracy of data collected from social media platforms and news websites, we employed data triangulation and consulted various sources to address this limitation. Furthermore, as the majority of our data were collected from online surveys, we recognize that impact response bias may influence our results. The issues with the cell phone network, internet, and power outages in the country have made it challenging to reach faculty members or students in certain areas of study. Additionally, in regions affected by ongoing conflict, we anticipate that attacks and the full extent of the damage may be underreported. Nonetheless, the difficulties in conducting on-site assessments in conflict-affected regions emphasize the importance of utilizing all available resources to gather data.

Another notable point is that the study did not address certain aspects that merit further investigation, such as the obstacles to continuing the educational process, as well as the viewpoints of both students and teachers regarding appropriate methods.

## Conclusion

To our knowledge, this study was the first to explore attacks on medical schools in Sudan amidst the ongoing war. The findings revealed that, across three months of conflict, 58.6% of the medical schools were ambushed, with the majority being looted or undergoing conversion into military bases. Despite this, 60% of the affected institutions were able to make efforts toward restoring their educational processes. To address the situation effectively, we recommend that the Ministry of Higher Education conduct a comprehensive on-ground assessment to determine the extent of the damage and estimate the cost needed for rebuilding the system. This would be a first step toward re-establishing the national educational landscape, ensuring the continuity of medical training, and minimizing the adverse impact of warfare on the healthcare system.

### Electronic supplementary material

Below is the link to the electronic supplementary material.

Supplementary Material 1

## Data Availability

The datasets analyzed during the current study are available from the corresponding author upon reasonable request.

## Abbreviations

SMC: Sudan Medical Council
HRH: Human Resources for Health
