# Supplementary material for: War and education: the attacks on medical schools amidst ongoing armed conflict, Sudan 2023
Source: Confl Health. 2024 Mar 29;18:23. doi: 10.1186/s13031-024-00584-7 (PMC10979604; doi:10.1186/s13031-024-00584-7)
Supplement: Supplementary file 1 — Supplementary Material 1 [file 13031_2024_584_MOESM1_ESM.docx]

**Table 1: Rate and methods of continuance of education in faculties**

| **Variable** | **Items** | **n** | **%** |
| --- | --- | --- | --- |
| Did the college propose any method to continue the educational process? (n=58) | Yes | 35 | 60.3 |
|  | No | 18 | 31.0 |
|  | No information | 5 | 8.6 |
| Methods used to continue the educational process (n=35) | On campus education | 1 | 2.8 |
|  | Online Education | 17 | 48.6 |
|  | Through collaboration with other universities outside/inside Sudan | 3 | 8.6 |
|  | Both | 14 | 40.0 |
